# Dihydrofolate Reductase I164L Mutation in *Plasmodium falciparum*, Madagascar 

**DOI:** 10.3201/eid1407.071498

**Published:** 2008-07

**Authors:** Didier Ménard, Valérie Andriantsoanirina, Martial Jahevitra, Céline Barnadas, Magali Tichit, Christiane Bouchier, Carol Hopkins Sibley

**Affiliations:** * Institut Pasteur, Antananarivo, Madagascar; † Institut Pasteur, Paris, France; ‡ University of Washington, Seattle, Washington, USA

**Keywords:** Plasmodium falciparum, sulfadoxine-pyrimethamine, drug resistance, dihydrofolate reductase, dhfr, I164L, Madagascar, letter

**To the Editor:** Malaria remains a major public health problem and a primary cause of illness in Madagascar (*1*). Since 2005, the National Malaria Control Program has revised its treatment policy and replaced chloroquine (CQ) with artesunate plus amodiaquine as first-line therapy for uncomplicated malaria and CQ with sulfadoxine-pyrimethamine (SP) for prevention of malaria during pregnancy. The latter choice was partially supported by high effectiveness of SP and absence of pyrimethamine resistance in Madagascar, in contrast to proximal African countries such as the Comoros Islands (*2*,*3*).

Analysis of the molecular basis of antimalarial drug resistance has demonstrated that mutations in the dihydrofolate reductase (*dhfr*) and dihydropteroate synthase genes are associated with development of SP resistance. It has been assumed that pyrimethamine resistance conferred by multiple mutations arose through stepwise selection of the S108N single mutant (except for the A16V/S108T allele). This single-point mutation decreases the sensitivity of *dhfr* to pyrimethamine in vitro by ≈10× (*4*). Subsequent mutations, such as N51I and C59R, cause additional decreases in the sensitivity of *dhfr* to pyrimethamine. Parasites with a triple-mutant allele (51I/59R/108N) are less sensitive to pyrimethamine in vitro, and patients infected with these parasites have a high probability of not responding to SP treatment (*5*).

Addition of I164L to 51I/59R/108N creates a quadruple-mutant allele and decreases the sensitivity of *dhfr* by ≈1,000× (*4*), eliminating the clinical effectiveness of SP, as observed in Southeast Asia and South America. However, the situation in Africa seems to be different because most studies conducted since the mid 1990s have shown the quadruple mutant to be rare, even in areas of intensive pyrimethamine use (*6*). Increasing SP resistance is principally a result of rapid selection for parasites that carry a triple-mutant allele that arose in Southeast Asia and has spread widely in Africa (*7*,*8*).

In 2006, blood samples were obtained from 114 children 6 months to 15 years of age enrolled in a clinical trial monitoring the efficacy of SP in treatment of uncomplicated *Plasmodium falciparum* malaria. The *dhfr* gene from pretreatment samples was sequenced at the Genomics Platform of the Pasteur Institute in Paris, France. Four (3%) samples contained the 108N single-mutant allele, 37 (32%) contained the 51I/59R/108N triple-mutant allele, and 1 (<1%) contained the I164L single-mutant allele. This latter allele was obtained from the blood of a 15-year-old girl from Ejeda in southern Madagascar. At enrollment in the trial, she had an axillary temperature of 37.8°C and a *P*. *falciparum* asexual parasite count of 74,880/μL. She was treated with the standard SP regimen (25 mg/kg sulfadoxine and 1.25 mg/kg pyrimethamine as a single dose on day 0). On the basis of the World Health Organization 2003 protocol (*9*), early treatment failure was noted on day 2, when the patient had signs of malaria with a temperature of 40°C and a parasite count of 770/μL. She was successfully retreated with a rescue regimen (quinine, 8 mg base/kg, 3 times a day for 7 days).

To confirm detection of the I164L allele, parasite DNA was extracted from blood spots obtained on days 0, 1, and 2 and sequenced. DNA templates were sent to a second independent laboratory (Department of Genome Sciences, University of Washington, Seattle, WA, USA) to rule out misidentification or polymerase errors. Nucleotide sequences obtained were compared with wild-type sequence (isolate 3D7, pyrimethamine-sensitive, GenBank accession no. NC_004318.1) by using BioEdit software (www.mbio.ncsu.edu/BioEdit/BioEdit.html). No point mutations other than I164L were observed in any samples obtained on 3 consecutive days. The nucleotide sequence determined in this study has been deposited in the GenBank database (accession no. EU280750).

Our report provides new insights into development of antifolate-resistant malaria and supports the findings of McCollum et al. (*10*) that the mutations in the *dhfr* gene do not always occur in a predictable, ordered, stepwise manner. Although the I164L allele was detected in a clinical sample from a patient who showed early treatment failure, blood levels of the drug were not measured. Thus, it is not possible to establish a causal connection between the I164L mutation and SP resistance. Moreover, the substantial reduction in parasite counts observed on days 0, 1, and 2 implied that SP treatment helped reduce parasite counts.

Our data confirm that the I164L mutation is beginning to appear in Africa. This observation highlights the risk for emergence and spread of the 51I/59R/108N/164L quadruple-mutant *dhfr* genotype by recombination between the I164L genotype and the 51I/59R/108N triple-mutant genotype, which would make SP widely ineffective for intermittent treatment of malaria during pregnancy. Further studies are needed to understand whether the I164L genotype has emerged in Madagascar because of local evolutionary history or has been introduced by gene flow from India or Southeast Asia, and to evaluate the risk for spread of this genotype to the rest of Africa through Madagascar.
